# Clinical features of BK-polyomavirus and cytomegalovirus co-infection after kidney transplantation

**DOI:** 10.1038/s41598-020-79799-6

**Published:** 2020-12-29

**Authors:** Ulrich Jehn, Katharina Schütte-Nütgen, Joachim Bautz, Hermann Pavenstädt, Barbara Suwelack, Gerold Thölking, Stefan Reuter

**Affiliations:** 1 Division of General Internal Medicine, Nephrology and Rheumatology, Department of Medicine D, University Hospital of Münster, 48149 Münster, Germany; 2 Department of Internal Medicine and Nephrology, University Hospital of Münster Marienhospital Steinfurt, 48565 Steinfurt, Germany

**Keywords:** Nephrology, Kidney diseases

## Abstract

BK polyomavirus (BKPyV) and cytomegalovirus (CMV) are the main viral pathogens affecting the graft and recipient outcome after allogenic kidney transplantation. It has recently been found that infection with both viruses has a greater impact on kidney graft function than a single infection. We retrospectively analyzed a cohort of 723 recipients who received kidney transplantation between 2007 and 2015 after living and postmortal donation for differences in risk and outcome parameters regarding BKPyV (DNAemia) and CMV (CMV DNAemia) co-infection compared to sole viremias and to patients without viremia. Of all kidney allograft recipients in our cohort, 8.2% developed co-infection with BKPyV DNAemia and CMV DNAemia, 15.1% showed BKPyV viremia alone and 25.2% sole CMV DNAemia. Acute rejection was closely linked with co-infection (multivariable analysis, p = 0.001). Despite the fact that the estimated glomerular filtration rate of patients with co-infection was noticeably reduced compared to patients with BKV or CMV infection alone, transplant survival and patient survival were not significantly reduced. Co-infection with BKPyV and CMV in kidney transplanted patients is significantly associated with inferior allograft function. Since co-infection is strongly associated with acute rejection, co-infected individuals should be considered a risk collective.

## Introduction

BK polyomavirus (BKPyV) and cytomegalovirus (CMV) infections are major infectious complications after allogenic kidney transplantation (KTx)^1,2^. Transplant immanent immunosuppressive therapy leads to (re-)activation of these common pathogens, which are normally latent in healthy individuals, so that they can cause a clinically relevant primary infection or, in most cases, re-activation.

Both viral infections can manifest as viremia or lead to an invasive organ disease. CMV disease may occur as pneumonitis, colitis, retinitis, myelosuppression and/or nephritis^3^, while BKPyV mainly affects the kidney allograft itself, causing tubulointerstitial nephritis^4^. Although the number of patients with CMV or BKPyV infections is high, patients who develop both infections/co-infections (CMV-BKPyV) are rare.

A CMV-positive donor, rejection episodes and deceased donor transplantation depict major risk factors for CMV infection in KTx patients^5^. The main risk factors for BKPyV infection are transplantation of grafts from BKPyV-seropositive donors into BKPyV-seronegative recipients, the number of HLA mismatches, ischemic injury, and prior acute rejection^6^. There is a controversial discussion whether CMV prophylaxis promotes BKPyV infection or not^7–10^. The exact relationship between CMV and BKPyV still lacks data, but it was suggested that both viruses are possible risk factors for each other^11^. Recently, Blazquez-Navarro et al. found in a large multicenter-study that CMV was associated with BKPyV and Epstein-Barr virus (EBV) within one year after KTx^12^. The authors observed that a combined CMV-BKPyV infection, regardless of which virus was first detected, resulted in reduced graft function 1 year after KTx, even at low viral load. Therefore, they concluded that the combined reactivation of CMV-BKPyV is a relevant complication of the post-transplant period with an unfavorable prognosis^12^. Since there is only very limited data about CMV-BKPyV infections after KTx and since there is no log-term data beyond the first post-transplant year available, we conducted the present study.

## Results

Baseline characteristics of the study populations are presented in Table 1. Mean age at transplantation was 52.0 ± 14.0 years, 60.4% were male, and 28.6% received a living donor transplantation.

**Table 1:** Patients’ demographic and clinical characteristics at transplantation.

Variable	All	No viremia	CMV DNAemia without BKPyV viremia	BKPyV DNAemia without CMV DNAemia	Both CMV and BKPyV DNAemia	p value
**Patients (n)**	723	380 (52.6%)	182 (25.2%)	102 (14.1%)	59 (8.2%)	** < 0.001**^**a**^
**Age at KTx (years)**	52.0 ± 14.0	50.9 ± 13.8	54.1 ± 14.5	50.5 ± 14.0	55.6 ± 13.0	** < 0.005**^**a**^
**Sex, male (n)**	437 (60.4%)	231 (60.8%)	101 (55.5%)	68 (66.7%)	37 (62.7%)	**0.290**^**b**^
**Mismatch-HLA-A**						**0.094**^**b**^
**None**	253 (35%)	140 (36.8%)	69 (37.9%)	32 (31.4%)	12 (20.3%)	
**1**	345 (47.7%)	171 (45.0%)	85 (46.7%)	56 (54.9%)	33 (55.9%)	
**2**	122 (16.9%)	68 (17.9%)	27 (14.8%)	13 (12.7%)	14 (23.7%)	
**Mismatch-HLA-B**						**0.499**^**b**^
**None**	168 (23.2%)	86 (22.6%)	43 (23.6%)	27 (26.5%)	12 (20.3%)	
**1**	344 (47.6%)	179 (47.1%)	90 (49.5%)	51 (50%)	24 (40.7%)	
**2**	208 (28.8%)	114 (30.0%)	48 (26.4%)	23 (22.5%)	23 (39%)	
**Mismatch-HLA-DR**						**0.302**^**b**^
**None**	185 (25.6%)	100 (26.3%)	43 (23.6%)	28 (27.5%)	14 (23.7%)	
**1**	344 (47.6%)	175 (46.1%)	87 (47.9%)	56 (54.9%)	26 (44.1%)	
**2**	208 (28.8%)	104 (27.4%)	51 (28.0%)	17 (16.7%)	19 (32.2%)	
**PRA > 85% (n)**	17 (2.4%)	10 (2.6%)	5 (2.8%)	0 (0%)	2 (3.4%)	**0.299**^**b**^
**PRA > 5% (n)**	75 (10.4%)	50 (13.1%)	27 (15.1%)	10 (9.8%)	9 (15.3%)	
**Living donor Tx (n)**	207 (28.6%)	127 (33.4%)	34 (18.7%)	31 (30.4%)	15 (25.4%)	**0.005**^**b**^
**ABO incompatible Tx (n)**	41 (5.7%)	25 (6.6%)	9 (4.9%)	5 (4.9%)	2 (3.4%)	**0.808**^**b**^
**Cold ischemia time (hours)**	8.3 ± 5.1	8.1 ± 5.2	8.6 ± 4.6	8.5 ± 5.2	8.3 ± 5.1	**0.475**^**a**^
**Warm ischemia time (minutes)**	33.0 ± 8.3	32.9 ± 8.4	33.3 ± 7.9	32.3 ± 7.4	34.0 ± 10.1	**0.722**^**a**^
**Dialysis prior to Tx (n)**	671 (92.8%)	350 (91.4%)	171 (95.5%)	94 (92.2%)	56 (94.9%)	**0.423**^**b**^
**Time on dialysis (months)**	57.4 ± 41.6	59.4 ± 45.0	58.2 ± 38.0	52.9 ± 38.6	53.5 ± 33.2	**0.622**^**a**^
**Previous Tx (n)**	94 (13%)	47 (12.4%)	29 (16.2%)	11 (10.8%)	7 (11.9%)	**0.540**^**b**^
**CMV mismatch D/R**						** < 0.001**^**c**^
D^−^/R^−^	125 (17.3%)	94 (24.7%)	5 (2.7%)	24 (23.5%)	2 (3.4%)	
D^−^/R^+^	125 (17.3%)	64 (16.8%)	33 (18.1%)	20 (19.6%)	8 (13.6%)	
D^+^/R^−^	170 (23.5%)	70 (18.4%)	57 (31.3%)	24 (23.5%)	19 (32.2%)	
D^+^/R^+^	302 (41.8%)	151 (39.7%)	87 (47.8%)	34 (33.3%)	30 (50.8%)	
**Induction therapy**						**0.960**^**c**^
No induction therapy	25 (3.5%)	11 (2.9%)	8 (4.5%)	4 (3.9%)	2 (3.4%)	
Basiliximab induction (n)	604 (83.5%)	319 (83.9%)	150 (82.4%)	86 (84.3%)	49 (83.1%)	
Anti-lymphocyte globulin (n)	37 (5.1%)	16 (4.2%)	12 (6.6%)	5 (4.9%)	4 (6.8%)	
Alemtuzumab (n)	14 (1.9%)	7 (1.8%)	3 (1.6%)	2 (2.0%)	2 (3.4%)	
Eculizumab (n)	2 (0.3%)	2 (0.6%)	0 (0%)	0 (0%)	0 (0%)	
Rituximab (n)	41 (5.7%)	25 (6.6%)	9 (4.9%)	5 (4.9%)	2 (3.4%)	
**Initial steroid use**	712 (98.5%)	374 (98.4%)	180 (98.9%)	101 (99.0%)	57 (96.6%)	**0.638**^**b**^
**Initial MMF use**	695 (96.1%)	361 (95.0%)	179 (98.3%)	97 (95.1%)	58 (98.3%)	**0.204**^**b**^
**Initial CyA use**	24 (3.3%)	19 (5%)	2 (1.1%)	3 (2.9%)	0 (0%)	**0.049**^**b**^
**Initial tacrolimus use (n)**	699 (96.7%)	361 (95%)	180 (98.9%)	99 (97.1%)	59 (100%)	**0.049**^**b**^
**Initial mTOR inhibitor use(n)**	29 (4.0%)	19 (5.0%)	4 (2.2%)	5 (4.9%)	1 (1.7%)	**0.383**^**b**^
**Diagnosis of ESRD, (n)**						**0.770**^**c**^
Hypertension	58 (8.0%)	25 (6.6%)	16 (8.8%)	9 (8.8%)	8 (13.6%)	
Diabetes mellitus	43 (5.9%)	21 (5.5%)	13 (7.1%)	6 (5.9%)	3 (5.1%)	
Polycystic kidney disease	105 (14.5%)	61 (16.1%)	20 (11.0%)	14 (13.7%)	10 (16.9%)	
Obstructive Nephropathy	35 (4.8%)	20 (5.3%)	8 (4.4%)	3 (2.9%)	4 (6.8%)	
Glomerulonephritis	231 (32%)	120 (31.6%)	58 (32.4%)	40 (39.2%)	13 (22%)	
FSGS	33 (4.6%)	16 (4.2%)	11 (6.0%)	2 (2.0%)	4 (6.8%)	
Interstitial nephritis	37 (5.1%)	18 (4.7%)	13 (7.1%)	4 (3.9%)	2 (3.4%)	
Vasculitis	23 (3.2%)	15 (3.9%)	4 (2.2%)	2 (2.0%)	2 (3.4%)	
Other	104 (14.4%)	54 (14.2%)	25 (13.7%)	14 (13.7%)	11 (18.6%)	
Unknown	54 (7.5%)	30 (7.9%)	14 (7.7%)	8 (7.8%)	2 (3.4%)	

*CMV* cytomegalovirus; *Tx* transplantation, *HLA* human leukocyte antigen, *PRA* panel reactive antibodies, *MMF* mycophenolate mofetil, *CyA* cyclosporine A, *mTOR* mechanistic target of rapamycin, *ESRD* end-stage renal disease, *FSGS* focal segmental glomerulosclerosis.

Bold: main variables and p-values.

^a^Kruskal–Wallis test.

^b^Fisher’s exact test.

^c^Chi square test.

Predominantly, a basiliximab-based induction therapy was used (84%), 5% of patients received anti-lymphocyte globulin (Table 1).

### Different constellations of DNAemia

**Figure 1:**
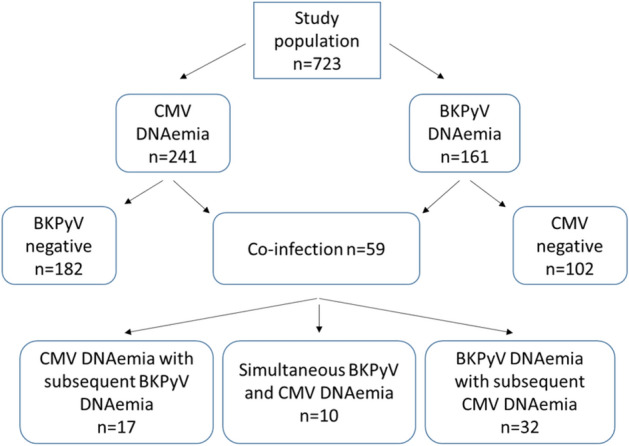
Overview of the different constellations of BKPyV and CMV viremia in our patient cohort.

For further analysis, the patient collective was divided into four subgroups, according to the constellation of CMV and BKPyV DNAemia (Fig. 1).

**Figure 2:**
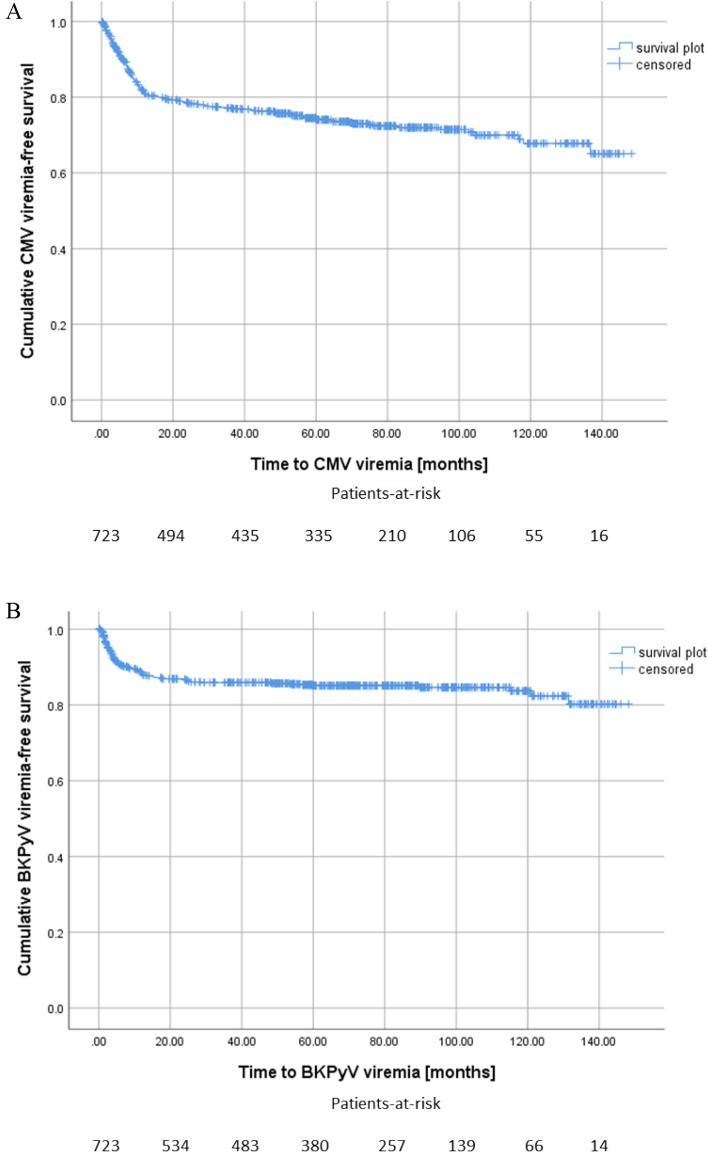

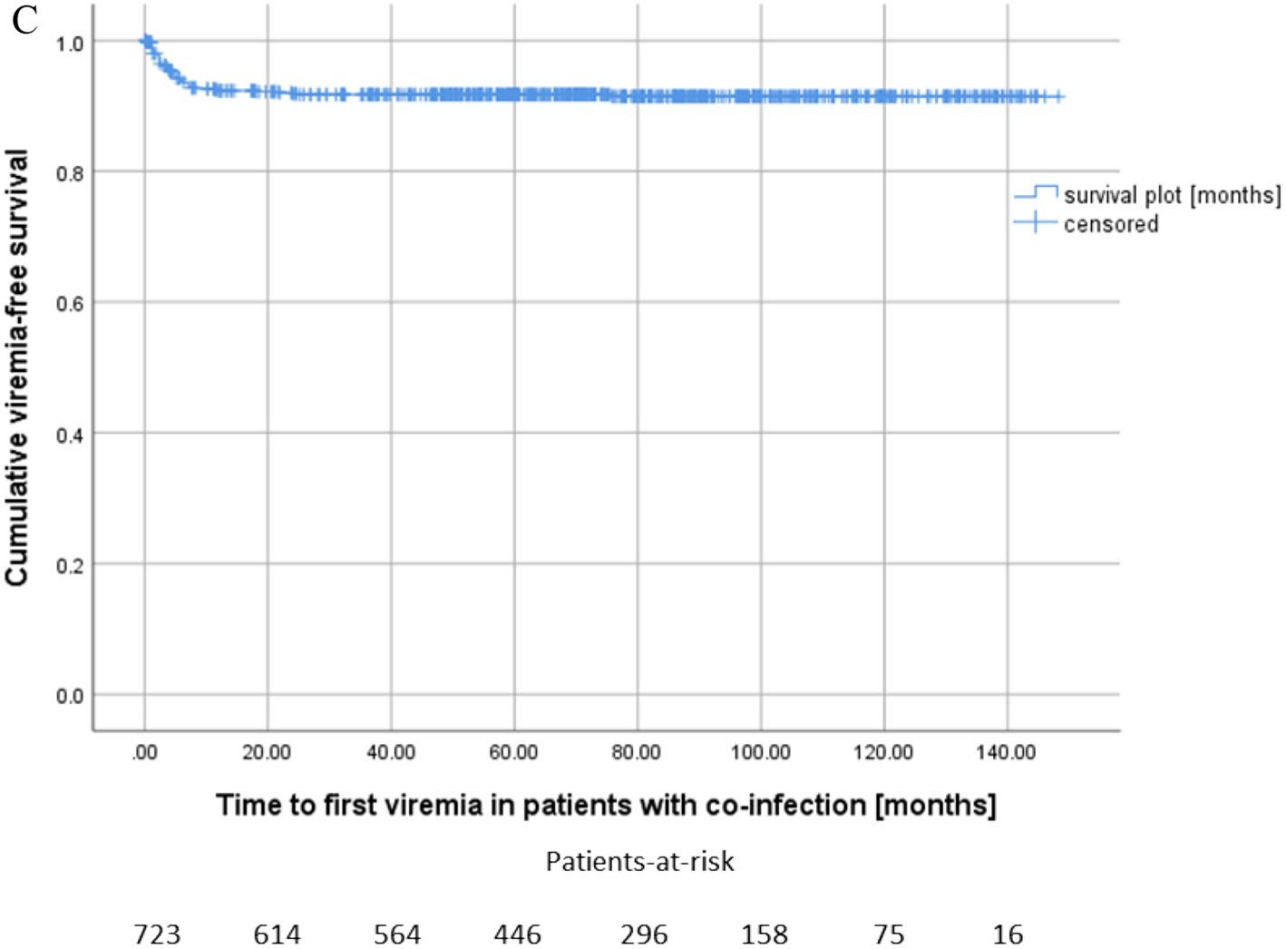
Kaplan Meier survival plots for onset of CMV viremia, median: 7.7 months. (**A**), BKPyV viremia, median: 4.0 months (**B**) and co-infection, median: 3.6 months (**C**) after kidney transplantation.

380 (52.6%) of the patients developed neither CMV nor BKPyV reactivation. 182 (25.2%) patients developed at least one episode of CMV DNAemia without BKPyV, 102 recipients (14.1%) one episode of BKPyV DNAemia without CMV DNAemia. The mean time until onset of isolated CMV DNAemia was 17.3 months (CI 95% 14.0–21.3), median 7.7 months, (CI 95% 6.7–8.6) and for isolated BKPyV DNAemia 12.5 months (CI 95% 7.9–17.1), median 4.0 months, (CI 95% 3.2–4.8), respectively. 59 (8.2%) of the patients showed co-infection with onset of CMV and BKPyV DNAemia over the course of the study, with a mean onset time of 6.3 months (CI 95% 3.6–9.0) and a median onset time of 3.6 months (IQR 3.84) for the first of the two diagnosed viremia (Fig. 2).

In allograft recipients with co-reactivation we observed a significantly shorter onset time of DNAemia compared to the onset of sole CMV or BKPyV. Interestingly, 45 (76.3%) co-infections occurred during the first six months after KTx, 54 (92%) co-infection occurred within the first 12 months after transplantation. Only one patient was diagnosed with co-infection beyond the second year after KTx. In contrast, 37 (19.8%) of sole CMV and 13 (12.7%) of sole BKPyV cases occurred after the second year (Fig. 2).

The cumulative incidence of sole CMV DNAemia at 1, 3, and 5 years was 19.4%, 25.2%, and 33.4%, respectively, resulting in an incidence of 5.5 CMV DNAemias per 100 person-years. The rate for BKPyV DNAemia alone at 1, 3, and 5 years was 12.3%, 15.8%, and 20.5%, respectively, resulting in an incidence of 3.1 BKPyV DNAemias per 100 person-years.

The cumulative incidence of CMV-BKPyV co-infection at 1, 3, and 5 years was 7.7%, 9.1%, and 11.5%, respectively, resulting in an incidence of 1.8 CMV-BKPyV infections per 100 person-years.

Taken together, 36.6% of patients with BKPyV DNAemia had a co-infection with CMV; conversely 24.5% of patients with CMV DNAemia also developed BKPyV DNAemia. In 32 (54.2%) of the 59 patients, BKPyV DNAemia occurred first, in 17 (28.8%) CMV DNAemia was diagnosed prior to BKPyV. In 10 patients (16.9%) both were diagnosed simultaneously (Fig. 1).

### Rejection episodes

Of all patients, 281 (38.9%) were diagnosed with at least one biopsy-proved acute rejection episode during the follow-up. Among them, 57 (20.3%) patients were diagnosed with antibody-mediated rejection (ABMR), 65 (23.1%) recipients with T-cell-mediated rejection (TCMR), 55 (19.6%) patients had a combined rejection and 100 (35.6%) were diagnosed with T-cell-borderline-rejection. Acute rejection was diagnosed by biopsy according to the BANFF criteria^14^. For statistical analysis of the rejection type, we only considered the first diagnosed rejection type, in those patients with more than one rejection episode. The treatment of acute rejection usually consisted of a steroid pulse for TCMR and T-cell-borderline rejection, followed by anti-lymphocyte globulin therapy for steroid-refractory TCMR. ABMR was treated with steroid pulse, plasmapheresis and intravenous immunoglobulins. CMV prophylaxis with valganciclovir and pneumocystis jirovecii prophylaxis with cotrimoxazole were administered for 3 months after rejection therapy.

**Figure 3:**
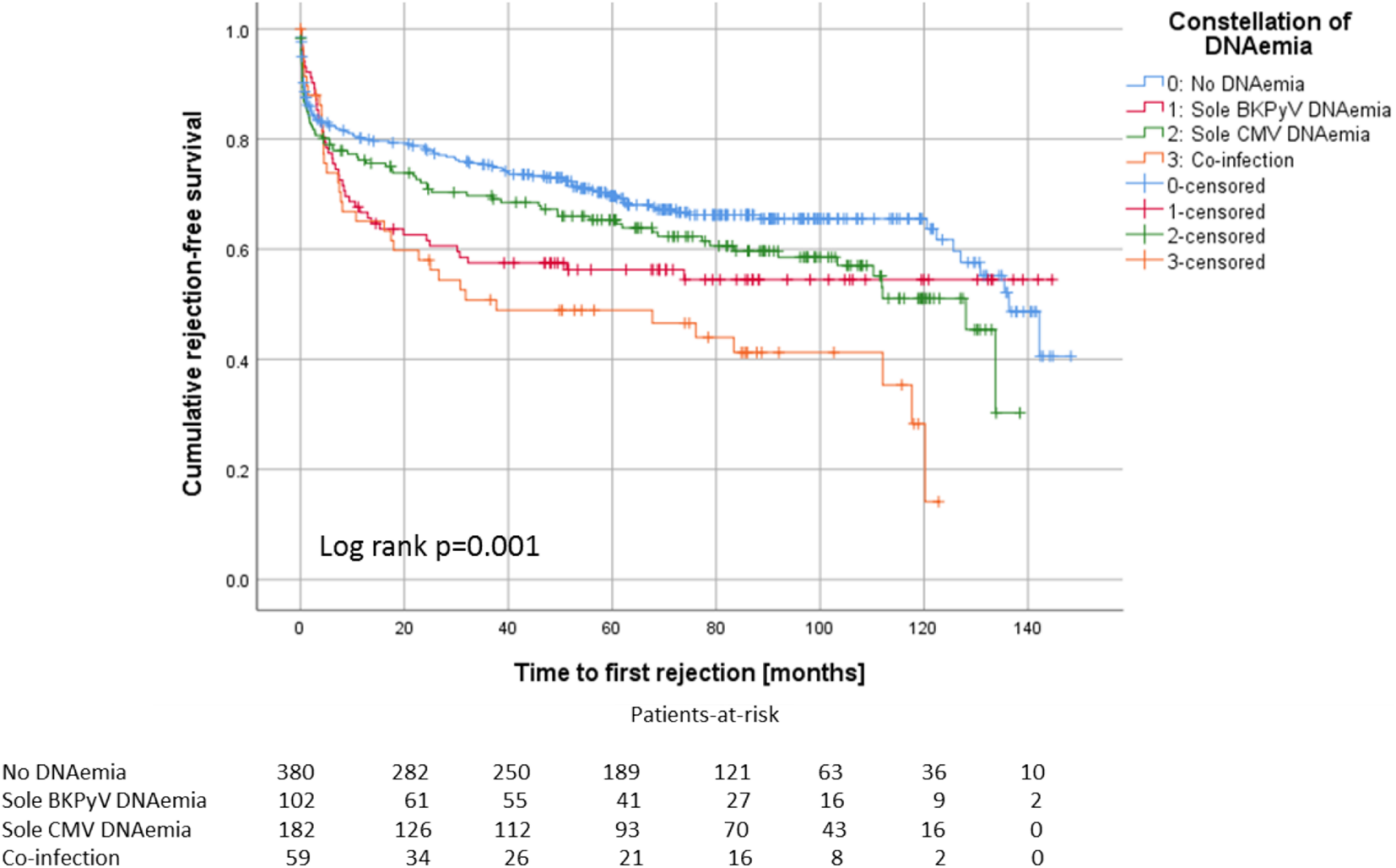
Kidney recipients who developed co-infection, showed the highest incidence of rejection episodes compared to those without viremia, BKPyV DNAemia or CMV DNAemia alone (Log-rank p = 0.001).

The proportion of patients with acute rejections was higher in the co-infection group (59.3%) than in the BKPyV^+^ group (44.1%) and the CMV^+^ group (41.5%) (p = 0.001, Fig. 3).

In the co-infection group, a total of 35 patients developed at least one rejection episode. Among these patients, in the majority of cases (22, 62.9%) viremia was diagnosed prior to rejection. In 10 cases (28.6%) viremia and rejection were diagnosed simultaneously, in only 4 cases (11.4%) rejection occurred ahead of viremia. Median onset time of the first acute rejection episode was 7.8 months (IQR 26.7).

### Renal function

**Figure 4:**
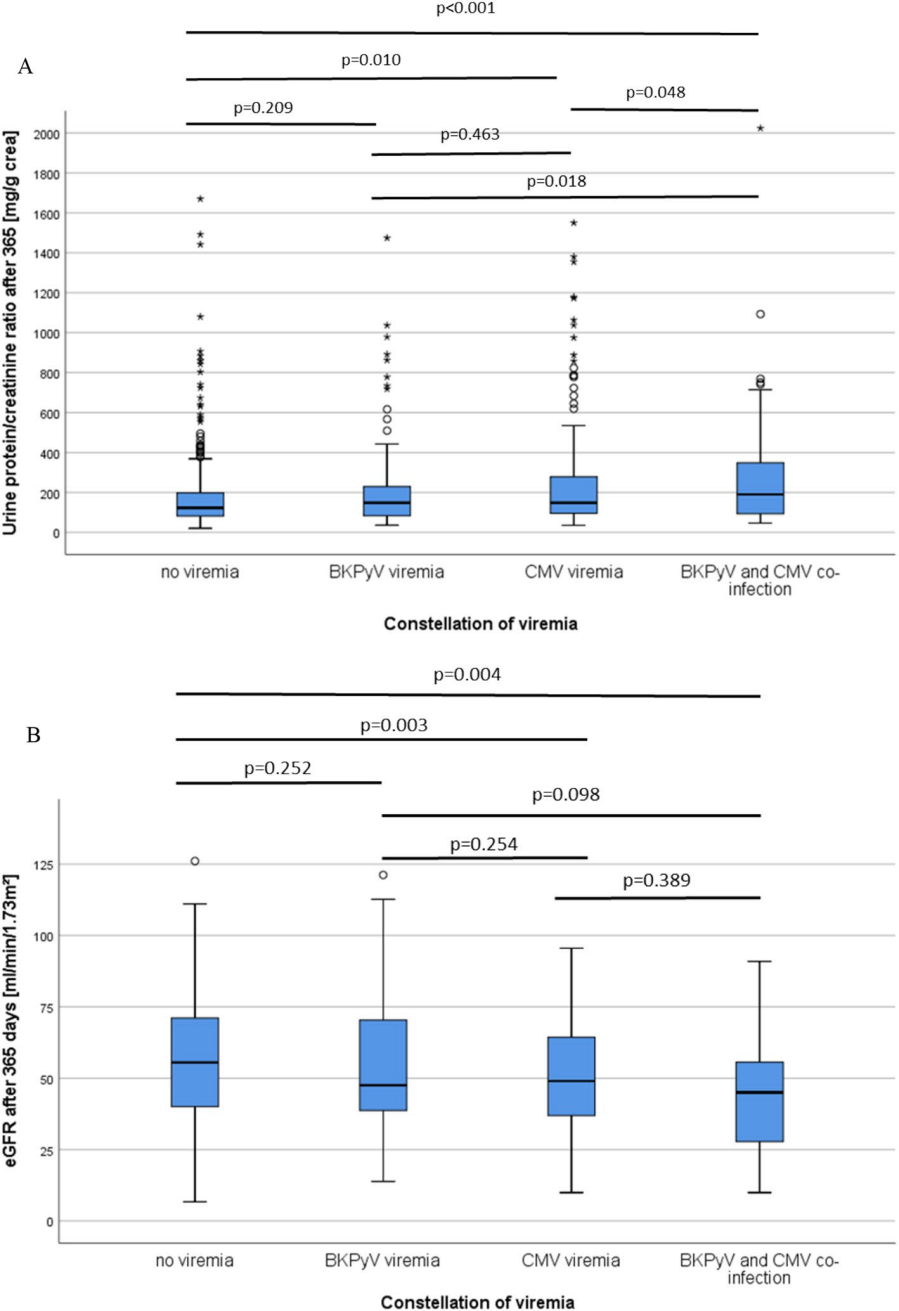
Transplant recipients who developed co-infection with BKPyV and CMV showed lower eGFR compared to recipients with isolated CMV or BKPyV DNAemia after 1 year (**A**) and also higher proteinuria (**B**). Patients without any viremia showed the highest eGFR levels and lowest proteinuria.

Patients with CMV and BKPyV co-reactivation showed not only a noticeably lower renal function (eGFR, each mean ± SD) after 1 (40.2 ± 11.1 ml/min/1.73 m^2^, Fig. 4A, Table 2), 3 (40.6 ± 15.8 ml/min/1.73 m^2^) and 5 years (42.9 ± 12.2 ml/min/1.73 m^2^) compared to patients without viremia (1 year 58.4 ± 19.3 ml/min/1.73 m^2^, 3 years 57.6 ± 19.0 ml/min/1.73 m^2^, 5 years 54.4 ± 19.7 ml/min/1.73 m^2^), but also compared to the CMV group (1 year 52.4 ± 19.6 ml/min/1.73 m^2^, 3 years 51.9 ± 20.8 ml/min/1.73 m^2^, 5 years 47.8 ± 20.8 ml/min/1.73 m^2^) and the BKPyV group (1 year 57.6 ± 24.2 ml/min/1.73 m^2^, 3 years 58.2 ± 27.2 ml/min/1.73 m^2^, 5 years 52.0 ± 28.8 ml/min/1.73 m^2^) (all p < 0.05). Patients with CMV DNAemia had noticeably lower eGFR levels compared to the BKPyV DNAemia group (p < 0.001), whereas eGFR levels of the BKPyV group were comparable to patients without viremia.

**Table 2:** Patients’ clinical outcome parameters.

Variable	No viremia (n = 380)	CMV DNAemia (n = 182)	BKPyV DNAemia (n = 102)	CMV and BKPyV DNAemia (n = 59)	p value
**Time until viremia, median (months), 1st, 3rd quartile**	–	7.7 (3.5,17.4)	4.0 (2.0,11.8)	3.6 (2.2,6.0)	
**DGF (n)**	80 (21.1%)	58 (31.9%)	23 (22.5%)	15 (25.4%)	**0.049**^**b**^
**eGFR at year 1, mean ± SD (ml/min/1.73 m**^**2**^**)**	58.4 ± 19.3	52.4 ± 19.6	57.6 ± 24.2	40.2 ± 11.1	**0.001**^**a**^
**eGFR at year 3, mean ± SD (ml/min/1.73 m**^**2**^**)**	57.6 ± 19.0	51.9 ± 20.8	58.2 ± 27.2	40.6 ± 15.8	**0.021**^**a**^
**eGFR at year 5, mean ± SD (ml/min/1.73 m**^**2**^**)**	54.4 ± 19.7	47.8 ± 20.8	52.0 ± 28.8	42.9 ± 12.2	**0.004**^**a**^
**UPCR at year 1, mean ± SD (mg/g crea)**	225 ± 200	155 ± 130	203 ± 318	279 ± 96	**0.003**^**a**^
**UPCR at year 5, mean ± SD (mg/g crea)**	455 ± 587	310 ± 320	245 ± 432	308 ± 361	**0.304**^**a**^
**Overall graft survival (mean, months) (95% CI)**	124.1 (119.0–129.2)	114.8 (108.2–121.4)	122.4 (113.4–131.5)	109.0 (95.4–122.8)	**0.834**^**c**^
NODAT* (n)	55 (14.5%)	38 (20.9%)	18 (17.6%)	6 (10.2%)	**0.109**^**b**^
**Pre-existent diabetes (n)**	44 (11.6%)	27 (14.8%)	9 (8.8%)	12 (20.4%)	**0.134**
**Highest CMV load, median (IU/ml), 1st, 3rd quartile**	–	1,100 (250,8163)	–	36,525 (8058, 428,323)	**0.687**^**d**^
**Highest BKPyV load, median (IU/ml), 1st–3rd quartile**	–	–	8565 (3,289,43,650)	75,885 (26,471, 98,900)	**0.898**^**d**^
**Rejection yes (n)**	126 (33.2%)	75 (41.2%)	45 (44.1%)	35 (59.3%)	**0.001**^**b**^
Antibody-mediated rejection	33 (8.7%)	3 (2.9%)	15 (10.4%)	6 (10.2%)	
T-cellular rejection	35 (9.2%)	8 (7.8%)	17 (9.3%)	5 (8.5%)	
Combined rejection	19 (5.0%)	8 (7.8%)	20 (11.0%)	8 (13.6%)	
T-cell-borderline rejection	39 (10.3%)	25 (24.5%)	20 (11.0%)	16 (27.1%)	

*DGF* delayed graft function, *eGFR* estimated glomerular filtration rate, *UPCR* urine protein/creatinine ratio, *NODAT* new onset diabetes after transplantation.

Bold: main variables and p-values.

^a^Kruskal–Wallis test.

^b^Fisher’s exact test.

^c^Log rank test.

^d^Mann–Whitney U test.

Proteinuria (each mean ± SD) was significantly elevated in the co-infection group after one year compared to the other groups (279 ± 96 mg/g crea, p = 0.003). The lowest urine protein/creatinine ratio (UPCR) was measured in the CMV group (155 ± 130 mg/g). In the group without viremia UPCR was 225 ± 200 mg/g crea, in the BKPyV group 203 ± 318 mg/g crea (Fig. 4B). After five years, there was no significant difference in UPCR between the groups (p = 0.304) (Table 2).

The median peak viral load of CMV and BKPyV DNAemia was higher in the co-infection group than in each group with a single virus infection. Nevertheless, due the high maximum viral loads, the difference did not reach statistical significance. High values > 500,000 IU/ml were graded as 500,000 IU/ml due to test kit limitations.

**Figure 5:**
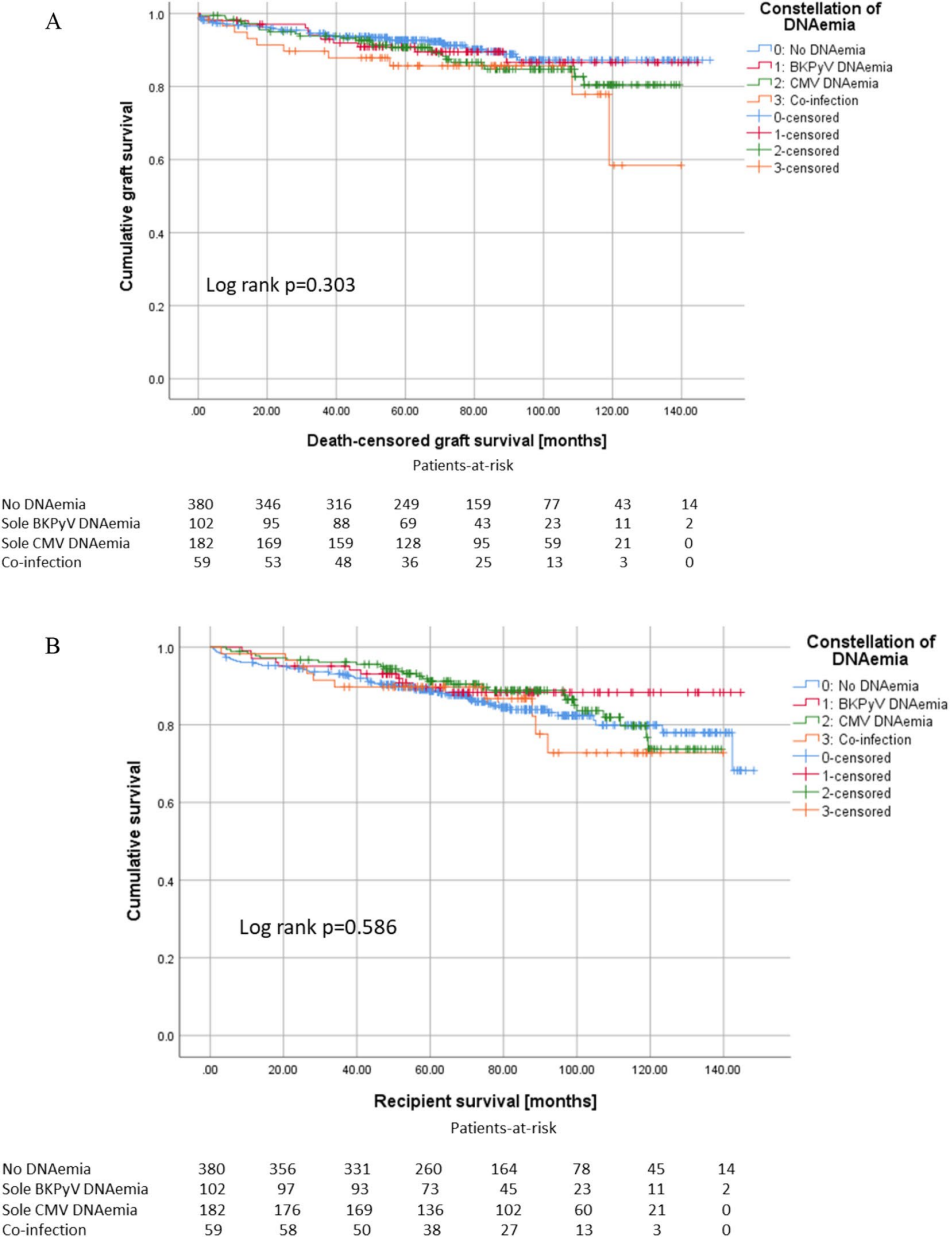
Co-infection with BKPyV and CMV does neither influence graft survival (**A**) nor recipients’ survival (**B**).

Patients with co-infection (mean 55.6 years) were 1.5 years older than patients with sole CMV DNAemia, 5.1 year older than patients with sole BKPyV DNAemia and 4.7 years older than patients without DNAemia. Kaplan–Meier analysis with Log rank testing did not show a noticeable difference for graft survival (p = 0.303, Fig. 5A) or recipients’ survival (p = 0.586, Fig. 5B) between the groups.

### Risk factors for patients with sole CMV or BKPyV DNAemia and co-infection

Risk factors for sole CMV DNAemia were CMV mismatch CMV D−/R+ (p < 0.001, HR 6.112), CMV D+/R− (p < 0.001, HR 8.449), CMV D+/R+ (p < 0.001, HR 6.916), postmortal donation (p < 0.001, HR 3.382), dialysis vintage (p = 0.017, HR 0.994) and delayed graft function (p = 0.003, HR 0.590) (Table 3).

**Table 3:** Cox regression for *CMV* DNAemia risk factors.

Variable	Hazard ratio	95% CI	p value
Age at KTx	0.995	0.980–1.010	**0.525**
Previous KTx	0.763	0.485–1.202	**0.243**
**CMV D**−**/R**− **(ref.)**			** < 0.001**
CMV D−/R+	6.112	2.367–15.786	** < 0.001**
CMV D+/R−	8.449	3.350–21.308	** < 0.001**
CMV D+/R+	6.916	2.782–17.193	** < 0.001**
Postmortal donation	3.382	1.743–6.561	** < 0.001**
Donor age	1.012	0.998–1.026	**0.099**
Time of dialysis	0.994	0.988–0.999	**0.017**
Cold ischemia time	0.957	0.913–1.003	**0.069**
Warm ischemia time	1.009	0.991–1.026	**0.332**
PRA	0.994	0.983–1.004	**0.218**
**No diabetes (ref.)**			**0.665**
NODAT	1.142	0.762–1.713	**0.519**
Preexisting diabetes	1.203	0.756–1.913	**0.435**
Delayed graft function	0.590	0.419–0.831	**0.003**
Initial Steroids	0.376	0.041–3.483	**0.389**
Initial MMF use	0.117	0.010–1.437	**0.094**
Initial CyA use	3.345	0.710–15.759	**0.127**
**Initial tacrolimus use**			.^**a**^
Inital mTor use	0.193	0.022–1.722	**0.141**
Acute rejections	0.790	0.573–1.089	**0.150**
HLA-mismatches	1.010	0.908–1.124	**0.855**

*KTx* kidney transplantation, *CMV* cytomegalovirus, *D/R* donor/recipient, *PRA* panel reactive antibodies, *NODAT* New onset diabetes after transplantation, *MMF* mycophenolate mofetil, *CyA* cyclosporine A, *mTOR* mechanistic target of rapamycin, *HLA* human leukocyte antigen.

Bold: p-values.

^a^Degree of freedom reduced because of constant or linearly dependent covariates.

For sole BKPyV DNAemia, acute rejection (p = 0.030, HR 0.626) and dialysis vintage (p = 0.008, HR 0.990) were noticeable risk factors in cox-regression analysis (Table 4).

**Table 4:** Cox regression for *BKPyV* DNAemia risk factors.

Variable	Hazard ratio	95% CI	p value
Age at KTx	1.001	0.982–1.020	**0.911**
Previous KTx	1.098	0.560–2.153	**0.786**
**CMV D−/R− (ref.)**			**0.626**
CMV D−/R+	1.217	0.649–2.282	**0.540**
CMV D+/R−	1.325	0.724–2.427	**0.362**
CMV D+/R+	0.956	0.547–1.627	**0.875**
Postmortal donation	2.208	0.927–5.258	**0.740**
Donor age	0.989	0.973–1.005	**0.184**
Time of dialysis	0.990	0.983–0.997	**0.008**
Cold ischemia time	0.996	0.938–1.057	**0.888**
Warm ischemia time	0.997	0.972–1.023	**0.827**
PRA	0.995	0.980–1.010	**0.484**
**No diabetes (ref.)**			**0.943**
NODAT	1.014	0.582–1.765	**0.962**
Preexisting diabetes	0.881	0.409–1.896	**0.746**
Delayed graft function	0.790	0.474–1.315	**0.364**
Initial steroids	0.398	0.042–3.784	**0.423**
Initial MMF use	0.474	0.011–21.274	**0.700**
Initial CyA use	2.506	0.572–10.976	**0.223**
**Initial tacrolimus use**			.^**a**^
Inital mTor use	0.274	0.006–12.856	**0.510**
Acute rejections	0.626	0.411–0.955	**0.030**
HLA-mismatches	1.010	0.877–1.163	**0.892**

*KTx* kidney transplantation, *CMV* cytomegalovirus, *D/R* donor/recipient, *PRA* panel reactive antibodies, *NODAT* New onset diabetes after transplantation, *MMF* mycophenolate mofetil, *CyA* cyclosporine A, *mTOR* mechanistic target of rapamycin, *HLA* human leukocyte antigen.

Bold: p-values.

^a^Degree of freedom reduced because of constant or linearly dependent covariates.

Thus, dialysis vintage is the only common risk factor for the onset of both types of viremia.

To test whether CMV DNAemia is a risk factor for BKPyV DNAemia, we performed a univariable binominal logistic regression analysis. For this analysis we had to include patients with co-infection into the BKPyV or CMV subgroup, respectively. Our data recently showed that CMV DNAemia was not a risk factor for BKPyV DNAemia or vice versa (p = 0.546, odds ratio 0.894)^5^.

For the occurrence of co-infections, the cox regression analysis revealed acute rejection as the most relevant risk factor (p = 0.001). Interestingly, in the majority of cases (62.9%) first viremia of co-infection preceded rejection. Only in 11.4% of cases rejection occurred first. In 28.6% of the cases, first viremia of co-infection and rejection were diagnosed simultaneously.

Regarding second viremia, 14 (40%) of the 35 co-infected patients who also showed acute rejection were diagnosed with second viremia prior to rejection, sixteen showed rejection prior to second viremia (45.7%). In five of the patients second viremia and first acute rejection episode were diagnosed simultaneously 14.3%).

Furthermore, a CMV-positive donor (CMV D+/R−, p = 0.008 and CMV D+/R+, p = 0.017) transplanted to a CMV-negative donor was identified as a risk factor (Table 5).

**Table 5:** Cox regression for *co-infection* risk factors.

Variable	Hazard ratio	95% CI	p value
Age at KTx	1.006	0.980–1.032	**0.670**
Previous KTx	1.320	0.550–3.166	**0.534**
**CMV D−/R− (ref.)**			**0.061**
CMV D−/R+	4.818	0.989–23.467	**0.052**
CMV D+ /R−	7.353	1.683–32.132	**0.008**
CMV D+ /R+	5.884	1.366–25.335	**0.017**
Postmortal donation	1.763	0.585–5.314	**0.314**
Donor age	1.011	0.985–1.037	**0.405**
Time of dialysis	0.996	0.987–1.006	**0.428**
Cold ischemia time	0.989	0.908–1.078	**0.805**
Warm ischemia time	1.012	0.980–1.045	**0.458**
PRA	1.010	0.997–1.023	**0.145**
**No diabetes (ref.)**			**0.228**
NODAT	0.488	0.202–1.178	**0.110**
Preexisting diabetes	1.122	0.537–2.344	**0.760**
Delayed graft function	1.092	0.584–2.043	**0.783**
Initial Steroids	2.400	0.384–15.014	**0.350**
Initial MMF use	0.751	0.004–130.437	**0.913**
Initial CyA use	177,281.905	0.000–4.335E+242	**0.965**
**Initial tacrolimus use**			.^**a**^
Initial mTor use	1.094	0.006–188.348	**0.973**
Acute rejection	0.399	0.232–0.689	**0.001**
HLA-mismatches	1.105	0.921–1.327	**0.281**

*KTx* kidney transplantation, *CMV* cytomegalovirus, *D/R* donor/recipient, *PRA* panel reactive antibodies, *NODAT* New onset diabetes after transplantation, *MMF* mycophenolate mofetil, *CyA* cyclosporine A, *mTOR* mechanistic target of rapamycin, *HLA* human leukocyte antigen.

Bold: p-values.

^a^Degree of freedom reduced because of constant or linearly dependent covariates.

## Discussion

Due to the increasing potency of immunosuppressive regimes, the rejection rate has decreased over the years, while susceptibility to infection has increased. In this work, the prevalence of the most important viral infections after KTx, namely CMV and BKPyV and combined CMV-BKPyV DNAemias are analyzed^2,15^. As there is still a lack of literature describing their interaction clinically after KTx, we have examined our large long-term study database based on a kidney transplantation program with an implemented structured monitoring for both viruses. Therefore, we are able to provide data beyond the first year after KTx.

We have identified CMV as the most common viral infection in our cohort, affecting almost one in three patients within 5 years of KTx. This is in line with data from the literature reporting incidences between 17 and 92% despite antiviral prophylaxis after KTx^15,16^. In contrast, Blazquez-Navarro et al. recently found a superiority of BKPyV infection over CMV from the epidemiological point of view^12^. However, this could be related to the short study period, since in contrast to BKPyV, CMV DNAemia occurs more frequently beyond the first year after KTx^5,17^. Every 5th patient developed BKPyV DNAemia only, while both infections occurred in about every 9th patient. In contrast, half of our patients had no CMV or BKPyV replication at all. Our data on single virus infections are broadly comparable with the data published in recent studies e.g. the MPA/TAC arm of the ATHENA study^18^. As mentioned above, data about CMV-BKPyV DNAemia is sparse but co-infection seems to range between 5 and 11% within 12 months after KTx^12,19^.

It was already suggested by Toyoda et al. in 2005 that both viruses are possible risk factors for one another^11^. Interestingly, in vitro data suggest that CMV can induce polyomavirus amplification and we observed in a previous case control study that the CMV mismatch was a risk factor for BKPyV^20,21^. The same observation was made in our present study (Table 5). However, CMV (mismatch) is not yet an established risk factor for BKPyV^2^. Therefore, prospective studies are necessary. In our cohort, CMV and BKPyV DNAemia were not associated, and the identified risk factors for CMV DNAemia alone differ from those for sole BKPyV DNAemia^5^. The dialysis vintage is the only one of our tested risk factors both infections (Tables 3, 4). In contrast, Elfadawy et al. observed that CMV DNAemia can indirectly protect against a later BKPyV DNAemia. The authors speculate that this is most likely due to an infection-related reduction of immunosuppression^19^. The same finding was observed the other way round—BKPyV infection protects against a later CMV infection^22^. Recently, Blazquez-Navarro et al. were unable to detect a clear temporal pattern of viral infections in their KTx cohort as 45.6% developed BKPyV before CMV DNAemia, and 33.3% had CMV before BKPyV hinting towards the before mentioned infection-related reduction of immune suppression^12^. The same was observed in our cohort. In addition, one should keep in mind that an increased intensity of immune suppression is an important risk factor for virus activation, and T-cell competence is necessary to permanently eliminate these infections^2,23–25^. In line with this, we identified the occurrence of acute rejection as a risk factor for CMV-BKPyV co-infection (Table 5). As previously argued and as seen by others, virus reactivation might occur due to increased immunosuppression caused by rejection therapy. Nevertheless, in the majority of patients, who suffered from both co-infection and rejection, viremia preceded rejection. Even regarding the occurrence of second viremia our data does not bring up an unambiguous relation between viremia and rejection episodes (and subsequent immunosuppressive therapy). 40% of the patients who suffered from both co-infection and acute rejection were diagnosed with second viremia prior to rejection, 45.7% showed rejection prior to second viremia, in 14.3% of the subjects second viremia and first acute rejection episode were diagnosed simultaneously. Therefore, these results cannot elucidate whether the difference in acute rejection detected may represent be a consequence or a cause of these co-infections. They suggest that either direction can be true.

This leads to the conclusion, that one the one hand viremia causes an activation and modulation of the immune system, which might be enhanced by double-viremia^26,27^, and on the other hand, that reduced immunosuppression as a treatment approach against viremia promotes the occurrence of rejection episodes. Blazquez-Navarro et al. could not confirm the correlation between co-infection and rejection in their study, so that it ultimately remains unclear^12,28^. Based on personal experiences and guidelines, we advise physicians to pay attention to virus reactivations or to prescribe prophylaxis in these cases (at least for CMV)^2,25^.

Somehow counterintuitive in this context is that the initial immunosuppression was not related to the frequency of coinfections in our patients (Tables 1, 5). This could be explained by the fact that most patients initially received a tacrolimus-based immunosuppression with too low numbers of alternative immunosuppressants in our study cohort. Other trials have shown that e.g. an mTOR-(mechanistic target of rapamycin) inhibitor containing regimen can protect from single virus infections, but studies do not provide coinfection rates^18,29^. Tacrolimus was also not associated to coinfection in the study by Blazquez-Navarro et al. (but trough level was associated to CMV DNAemia, also in the study of Elfadawy et al.^19^ and it was previously shown by us that a fast tacrolimus metabolism (need for higher tacrolimus doses) is linked to BKPyV activation^12,21,30^. As we did not analyze trough levels in the present cohort we cannot comment on this further.

Since, in contrast to the outbreak of a single CMV DNAemia (median 7.7 months after KTx), coinfection occurred earlier with a median of 3.6 months after KTx, while the single BKPyV DNAemia occurred 4.0 months after KTx (median), one can speculate that this fits into the concept of over-immunosuppression-related virus activation, since the immunosuppressive regime is more intense early after KTx than late.

A hitherto neglected point is that viral infections are known to modulate and dysregulate the immune system and are therefore able to trigger rejection episodes^31^. Further, rejection may be fostered by a release pro-inflammatory cytokines, upregulation of HLA or vascular adhesion molecules during CMV DNAemia and therefore require a further increase in immunosuppressive therapy^32–34^. However, due to the study design we were not able to analyze this complex interaction further.

Blazquez-Navarro et al. observed a significant negative impact of combined BKPyV and CMV (re-)activation even at low replications levels on renal allograft function 1 year after KTx^12^. Unfortunately, survival data according to the virus infection groups is not reported. In line with the data, Kaul et al. found in an analysis of tissue-invasive CMV-BKPyV co-infections in renal transplants biopsies that coinfected grafts had an inferior function. Interestingly, coinfection had no effect on patient and graft survival^35^. This is somehow surprising since an impact on allograft survival has been reported previously for single virus infections by others^15,36,37^. Nevertheless, this is not consistently observed and may be related to the time of the study, test and treatment strategies, immunosuppressive protocols and the extent of invasive infections^5,38^. As the absolute number of investigated biopsies was low in the study published by Kaul et al. it could be assumed that it was underpowered to detect survival effects (CMV, n = 17, BKPyV, n = 8; CMV-BKPyV, n = 12). However, we analyzed distinctly larger numbers, but were also unable to identify effects of CMV and BKPyV on graft and patient survival (Fig. 5^5,21^). A possible explanation for these observations could be that, for example, the absolute number of invasive infections such as BK nephropathy in our center is < 10% of patients with BKPyV DNAemia^21,30^. Nevertheless, in agreement with Blazquez-Navarro and Kaul et al. we observed that patients with co-infections had a worse renal graft function compared to non-infected or single virus infected patients^12,35^. Ultimately, we cannot know for certain whether the reduced graft function of patients with co-infection results from the co-infection itself or is biased by the more frequent occurrence of rejection episodes. Conspicuously, the decrease in eGFR in the coinfection group entirely takes place within the first year, with no significant subsequent further decline thereafter. In our understanding, events like rejections, infections or surgical problems frequently occur early after transplantation and therefore contribute to this finding. A second reason might be, that patients with severe loss of eGFR are not considered for subsequent eGFR-measurement, when this severe eGFR-loss led to terminal graft failure.

In this study, we decided to focus on events with any detectable DNAemia, instead of manifest disease since CMV disease or organ manifestation is sometimes difficult to diagnose and tricky to distinguish from other pathomechanisms, for example in cases of leukopenia, which might be drug-induced as well. A second reason was that indirect effects of CMV-infection^39^ would possibly be underestimated by only focussing on CMV disease.

A pitfall regarding BKPyV nephropathy is that a relevant number might not be confirmed by biopsy, hence this would not entail further therapeutical consequence in some cases. Moreover, as BKPyV nephropathy can occur focally, false negative results are possible.

Nevertheless, in some cases transient asymptomatic low-level DNAemia may not impact patient or allograft outcome, which could be an explanation for the lack of differences regarding patient or allograft survival in our cohort.

Our study has limitations. Since it is a retrospective analysis, the study is only suitable to generate hypotheses. Moreover, the study design does not allow to distinguish the effects of co-infection from those of rejection episodes associated with CMV-BKPyV. In addition, we neither analyzed trough levels nor drug dosages in the present study.

The connection between CMV and BKPyV has not been clearly clarified yet, as the data on CMV-BKPyV (re-)activation after KTx is limited. Therefore, our study is of interest because we provide a comprehensive analysis of risk factors and transplant outcome for CMV and BKPyV co-infections that occurred in more than 8% of kidney transplant recipients in a large European cohort with long-term follow-up.

Our study should stimulate the performance of prospective studies that investigate the complex interaction between transplant rejection and CMV-BKPyV after KTx. We further want to increase the awareness of transplant physician to this relationship, as it represents a considerable disease burden for the patients concerned.

## Methods

### Study design and population

We have re-analyzed the data from our previous CMV study, which focused on the risk factors for CMV after living and postmortal kidney donation^5^. We included 723 adult patients (follow-up of 3292 patient-years, median follow-up 6.4 years) who were transplanted at our center between 01/01/2007 and 12/31/2015. The demographic and clinical characteristics of the patients were recorded at the KTx. Written informed consent was obtained from all patients to record their data at the time of KTx. Data was taken from patients’ file and personal information was anonymized prior to the analysis. This study was performed in accordance with the Declaration of Helsinki and the International Conference on Harmonization Good Clinical Practice guidelines and approved by the local ethics committee (Ethik Kommission der Ärztekammer Westfalen-Lippe und der Medizinischen Fakultät der Westfälischen Wilhelms-Universität, 2014-381-f-N). The induction therapy was chosen according to the immunological risks of the patients (Table 1). The triple immunosuppressive standard regimen consisted of tacrolimus (6–10 ng/ml), mycophenolate mofetil and steroids (Table 1). eGFR was calculated using the CKD-EPI formula^13^.

### CMV and BKPyV testing and treatment

Valganciclovir was given for 100 days for D^+^/R^−^, D^+^/R^+^ and D^−^/R^+^ recipients, for 200 days for D ^+^/R ^−^ recipients beyond June 2009, and no valganciclovir in D^−^/R^−^.

Virus screening was performed monthly within the first 6 months after KTx, every second month during months 6–12, and on indication. EDTA-blood was used for the kPCR PLX CMV DNA-assay and the kPCR PLX BKV-assay, respectively, in combination with the VERSANT kPCR molecular system (Siemens Healthcare Diagnostics, Eschborn, Germany). QNAT threshold for CMV DNAemia was > 214.6 IU/ml and > 114 IU/ml for BKPyV DNAemia.

CMV-DNAemia was treated with antiviral medication, when organ manifestation or any kind of symptoms occured. Furthermore, asymptomatic CMV-DNAemia was treated above 500 IU/ml. In case of BKPyV-Dnaemia immunosuppressive therapy was adjusted when BKPyV nephropathy was diagnosed or at BKPyV DNAemia above 1000 IU/µl.

Findings below 500 IU/ml for CMV-DNAemia and 1000 IU/ml for BKPyV-DNAemia respectively were controlled every 2 weeks.

### Diagnosis of rejection

Only biopsy proven rejections were considered as relevant for our study. Kidney biopsy was performed if creatinine levels increased (≥ 0.3 mg/dl) and/or a significant proteinuria or new donor-specific antibodies occurred. The kidney biopsies were evaluated by two pathologists. Rejections were diagnosed by histological biopsy evaluation based on the BANFF classification.

### Statistical analysis

The data was analyzed with IBM SPSS Statistics 24 (IBM Corp., Armonk, New York, USA). The results are expressed as a mean with standard deviation, median with interquartile range (IQR), or number/percent. Non-continuous parameters were analyzed by Fisher’s exact test and chi-square test and continuous parameters were analyzed by Mann–Whitney U-test and Kruskal–Wallis test, respectively, if appropriate. A *p*-value below 0.05 was considered statistically noticeable.

The cumulative probability of developing CMV DNAemia, BKPyV DNAemia or co-infection in the kidney transplant cohort was calculated by Kaplan–Meier analysis and the curves were compared using the log-rank test. The cumulative incidences of the first CMV, BKPyV and CMV-BKPyV co-infection diagnosis at 1, 3 and 5 years of follow-up were calculated.

To evaluate risk factors for the onset of co-infection or each sole viremia, we performed multivariable cox-regression analyses.

A binominal logistic regression analysis was performed to clarify whether BKPyV DNAemia is a risk factor for CMV DNAemia or vice versa.
